# Analysis of the effects of M2 macrophage‐derived PDE4C on the prognosis, metastasis and immunotherapy benefit of osteosarcoma

**DOI:** 10.1111/jcmm.18395

**Published:** 2024-05-22

**Authors:** Feng Pan, Runsang Pan, Rui Hu, Hao Zhang, Shan Lei, Lu Zhang, Changhua Zhou, Zhirui Zeng, Xiaobin Tian, Quan Xie

**Affiliations:** ^1^ College of Big Data and Information Engineering Guizhou University Guiyang China; ^2^ Department of Bone and Joint Surgery Beijing Jishuitan Hospital Guizhou Hospital Guiyang China; ^3^ School of Basic Medicine Guizhou Medical University Guiyang China; ^4^ The 4th Department of Orthopaedics The Second People's Hospital of Jingmen Jingmen China; ^5^ College of Clinical Medicine Guizhou Medical University Guiyang China; ^6^ Postdoctoral Workstation Affiliated Hospital of Guizhou Medical University Guiyang China

**Keywords:** immunotherapy, metastasis, osteosarcoma, PDE4C, prognosis, tumour‐associated macrophage

## Abstract

Tumour‐associated macrophages (TAMs), encompassing M1 and M2 subtypes, exert significant effects on osteosarcoma (OS) progression and immunosuppression. However, the impacts of TAM‐derived biomarkers on the progression of OS remains limited. The GSE162454 profile was subjected to single‐cell RNA (scRNA) sequencing analysis to identify crucial mediators between TAMs and OS cells. The clinical features, effects and mechanisms of these mediators on OS cells and tumour microenvironment were evaluated via biological function experiments and molecular biology experiments. Phosphodiesterase 4C (PDE4C) was identified as a pivotal mediator in the communication between M2 macrophages and OS cells. Elevated levels of PDE4C were detected in OS tissues, concomitant with M2 macrophage level, unfavourable prognosis and metastasis. The expression of PDE4C was observed to increase during the conversion process of THP‐1 cells to M2 macrophages, which transferred the PDE4C mRNA to OS cells through exosome approach. PDE4C increased OS cell proliferation and mobility via upregulating the expression of collagens. Furthermore, a positive correlation was observed between elevated levels of PDE4C and increased TIDE score, decreased response rate following immune checkpoint therapy, reduced TMB and diminished PDL1 expression. Collectively, PDE4C derived from M2 macrophages has the potential to enhance the proliferation and mobility of OS cells by augmenting collagen expression. PDE4C may serve as a valuable biomarker for prognosticating patient outcomes and response rates following immunotherapy.

## 1 INTRODUCTION

Osteosarcoma (OS) is a bone cancer which usually occurs in long bones. The outcome of OS patients is radically poor, accounting for high rate of metastasis.^1^ Similarly, OS patients have a low response rate to immunotherapy, an emerging therapy account for the immunosuppressive microenvironment.^2^ Macrophages and their precursor monocytes are key parts of innate immune system.^3^ Previous studies indicated that macrophages in the tumour tissues, also named tumour associated with macrophages (TAMs), encompassing both M1 and M2 subtypes, contributed to the proliferation, metastasis and immunosuppression of cancers, including OS.^4^ However, the factors involved in the crosstalk between OS cells and TAMs were known to be limited.

Cyclic nucleotide phosphodiesterase (PDE) family is a super‐family involved in hydrolysing the second messenger cAMP. Therefore, PDEs participate in a series of signalling pathways of proliferation and mobility via regulating the cellular concentration of cAMP.^5^, ^6^ PDE4C is a member of PDE family, while high expression of PDE4C was observed in non‐small cell lung cancer tissues.^7^ Similarly, high expression of PDE4C was related to shorter survival days in glioma. Suppression of PDE4C increased the apoptosis of glioma cells.^8^ Regrettably, role of PDE4C in OS was still unknown.

The purpose of this study was to investigate key factors involved in crosstalk between OS cells and TAMs. We demonstrated that PDE4C derived from M2 macrophages has the potential to enhance the proliferation and mobility of OS cells by augmenting collagen expression. PDE4C may serve as a valuable biomarker for prognosticating patient outcomes and response rates following immunotherapy.

## 2 MATERIALS AND METHODS

### 2.1 Single‐cell RNA‐sequencing analysis

In the current study, the single‐cell RNA (scRNA) sequencing profile GSE162454 of OS tissues provided by Liu et al.^9^ was analysed in R software. The Seurat package^10^ was employed to eliminate genes expressed in less than 200 cells and cells with less than 200 features. Prior to perform PCA analysis, scRNA sequencing data was performed normalization, and centralized processing. The clustering process of cells utilized the top 2000 genes exhibiting the highest variability, with a resolution parameter of 0.5. Cell annotation was conducted in CellMarker 2.0 (http://bio‐bigdata.hrbmu.edu.cn/CellMarker/) utilizing the top 10 signature genes of each cell cluster. Following cell annotation, cell clusters were merged, and high‐abundance genes in each cell type were chosen based on a threshold of expression in 25% of the cell type and a 1.25‐fold change compared to the expression levels in other cells. Furthermore, the cell communication was analysed using scConnect package.

### 2.2 RNA‐sequencing data download and pro‐processing

Routine next‐generation sequencing data and clinical information used for analysis in the present study were downloaded from TARGET database ((https://ocg.cancer.gov/programs/target)) and GEO database (https://www.ncbi.nlm.nih.gov/geo/) with the index word as GSE39055, respectively. Total 85 OS patients with complete prognostic information were enrolled in TARGET program, while the number of patients in GSE39055 was 37. Prior to analysis, the RNA‐sequencing of these two cohorts was performed gene annotation and normalization.

### 2.3 OS tissues collection and immunohistochemical (IHC) staining

With the approval of the Human Ethics Committee of Guizhou Medical University (approve number: [2020]079), a total of 44 OS tissues were collected. Among them, 26 tissues were provided by patients diagnosed without metastasis, while 18 tissues were provided by patients diagnosed with metastasis. Patients enrolled in the study all obtained written informed consent. The process of IHC experiments was shown in our previous study.^11^ The dilution and catalogue number of primary antibodies used in the present study were shown as follows: PDE4C (1:200, catalogue: DF3872, Affinity, USA), CD206 (1:200, catalogue: 18704‐1‐AP, Proteintech, Wuhan, China), CD68 (1:1000, catalogue: ab303565, Abcam, USA) and PDL1 (1:5000, catalogue: 66248‐1‐Ig, Proteintech, Wuhan, China).

### 2.4 Cell culture and co‐culture condition

Human monocyte‐like cell THP‐1 was acquired from Procell (Wuhan, China) and cultured in the RMPI‐1640 medium with 10% FBS. Human bone mesenchymal stem cells (BMSCs), 143B and U2OS OS cell lines were obtained from ATCC (USA) and cultured in DMEM medium with 10% FBS. All cells were cultured in the 37°C environment with 5% CO_2_. A total of 200 ng/mL phorbol 12‐myristate 13‐acetate (PMA, MCE, Wuhan, China) was used for 3 days to induce monocyte‐like THP‐1 into macrophage‐like THP‐1 (also named M0). Then, IL‐4 (20 ng/mL, Abcam, USA) was used to translate M0 macrophage into M2 macrophage, while LPS (100 ng/mL, Abcam, USA) and IFN‐γ (20 ng/mL, Abcam, USA) were combined used to translate M0 macrophage into M1 macrophage. For constructing co‐culture conditions, transwells (Corning, USA) with 0.4 μm bore diameter were used. THP‐1 or macrophage cells were set in upper chambers, and 143B or U2OS cells were set in low chambers. After culturing for 48 h, 143B and U2OS were digested to perform further experiments.

### 2.5 Immunofluorescence

Cells were fixed in cell culture dish using 4% paraformaldehyde (Boster, Wuhan, China). Following the disruption of cell membranes by 0.3% Triton‐X reagent (Boster, Wuhan, China), 5% BSA (Boster, Wuhan, China) was used to perform blocking. Primary antibodies including CD68 (1:200, Catalogue: 28058‐1‐AP; Proteintech, Wuhan, China), CD11B (1:200, Catalogue: A1581; Abconal, Wuhan, China), α‐SMA (1:200, Catalogue: 55135‐1‐AP; Proteintech, Wuhan, China) and FAP (1:100, Catalogue: A6349; Abconal, Wuhan, China) were added overnight. After washing with PBS and incubating with secondary antibodies, cell nuclear was stained with DAPI reagent. The fluorescence signal was detected in a Zeiss‐inverted fluorescence microscope (Germany).

### 2.6 Exosome extraction

Exosomes were isolated from the cell culture medium using the Invitrogen™ Total Exosome Isolation Kit (ThermoFisher, USA). Specifically, 1 mL of the cell culture medium was combined with the precipitation reagent supplied by the kit, and the exosomes were subsequently isolated following the guidelines provided by the manufacturer. Following this, transmission electron microscopy and western blotting techniques were employed to confirm the morphology and marker proteins of the extracted exosomes. The exosomes were preserved at a temperature of −80°C prior to conducting cell culture experiments and molecular analyses.

### 2.7 RT‐qPCR

Total RNA in cells and tissues were obtained using TRIzol reagent. Rayscript cDNA synthesis kit (GENEray Biotechnology, Shanghai, China) was used to perform the cDNA synthesis. After adding SYBR green reagent (GENEray Biotechnology) and primers in cDNA solution, amplification and real‐time fluorescence detection were conducted. *GAPDH* was set as a loading control to measure the relative levels of target genes. Exosomal RNAs were isolated utilizing the exoRNeasy Serum/Plasma Maxi Kit (Yeasen Biotech, Shanghai, China), which had been pre‐enriched with 25 fmol of C. elegans cel‐miR‐39 standard RNA (Sangon, Shanghai, China). The *C. elegans* cel‐miR‐39 standard RNA served as a control to standardize the exosomal RNAs. Used primers were shown in Table S1.

### 2.8 Western blotting

Total protein in cells were extracted by a RIPA buffer (Future‐biotech, Nanjing, China). The 12.5% SDS‐PAGE gels (Acro Biosystems, Shanghai, China) were used for separating the proteins (30 μg/per line). Then, proteins were transferred onto PVDF membranes (Acro biosystems, Shanghai, China). After blocking with 5% skim milk powder, membranes were incubated with anti‐CXCL11 (1:2000, ab259863, Abcam, USA), anti‐ARG1 (1:3000, 16001‐1‐AP, Proteintech, Wuhan, China), anti‐PDE4C (1:500, 21754‐1‐AP, Proteintech, Wuhan, China), Calnexin (1:5000, 10427‐2‐AP, Proteintech, Wuhan, China), TSG101 (1: 4000, 28283‐1‐AP, Proteintech, Wuhan, China), HSP70 (1:5000, 10995‐1‐AP, Proteintech, Wuhan, China), COL11A2 (1:1000, ab227945, Abcam, USA), COL9A1 (1:1000, PA5‐93062, Invitrogen, USA), COL9A3 (1:1000, SAB2109132, Sigma‐Aldrich, USA) and anti‐GAPDH (1:5000, 60004‐1‐Ig, Proteintech, Wuhan, China) primary antibodies overnight. After washing three times with TBST, membranes were incubated with secondary antibodies and visualized using ECL reagent (Acro biosystems, Shanghai, China). GAPDH was set as loading control.

### 2.9 Cell transfection

*PDE4C* targeting and negative control (NC) small interfering RNA (siRNA) were acquired from GeneChem (Shanghai, China). The empty vector and complete length PDE4C cDNA were inserted into pCMV5 plasmids to create NC and PDE4C overexpression (PDE4C‐OE) plasmids. The transfection of siRNAs and plasmids was conducted using Lipo2000 reagent (Thermo Fisher Scientific, USA).

### 2.10 Cell proliferation detection

For the CCK‐8 method, cells were seeded in a 96‐well plate with a density of 2000 cells per well. After the specific points in time (cell adhesion, 24, 48 and 72 h), a total of 10 μL CCK‐8 reagent (Beyotime, Jiangsu, China) was added, and each well was detected at 450 nm. As for colony formation assay, cells were set in six‐well plates with 500 cells per well. After 12 days, cell colony was fixed followed by staining crystal violet (0.5%) for 15 min. Finally, PBS was used to wash plate and the number of colonies was counted.

### 2.11 Cell mobility detection

For wound healing assay, monolayer cells with confluence greater than 95% in a six‐well plate have created a wound. Following washing by PBS twice, a serum‐free DMEM medium was added. The wound condition was recorded at 0 and 24 h, while migration rates were determined based on the wound healing condition. Transwell with 0.8 bore diameter (Corning, USA) were pre‐coated matrigel to measure the invasion ability of OS cells. In brief, after co‐culturing, digesting and resuspending in 300 μL medium, a total of 2 × 10^5^ OS cells were set in upper chamber, while 700 μL medium containing 10% FBS was placed in the lower chamber. Following culturing by 48 h, cells were fixed and stained with 1% crystal violet. Rubbing out non‐invasion cells, the condition of transwell was recorded, and invasion ability of cells was calculated based on invasive cell number per field.

### 2.12 Profile merge and differentially expressed genes (DEGs) analysis

The gene expression profiles of TARGET and GSE39055 were merged and batch‐corrected via an R package ‘sva’. Then, the merged expression profiles were re‐ordered based on the expression of PDE4C. According to median expression value of the PDE4C level, OS samples were classed into high and low PDE4C subgroup. The DEGs between high and low PDE4C group OS tissues were analysed by Limma package using the threshold as adjust *p* value <0.05 and |log2 fold change| ≥1.

### 2.13 Enrichment analysis

Enrichment analyses containing GO analysis and KEGG analysis were performed in online tool Sanger Box (http://vip.sangerbox.com/home.html) based on the reference from DAVID (https://david.ncifcrf.gov/). The top 5 terms with *p* < 0.05 were exhibited in circle diagram.

### 2.14 TIDE analysis

For predicting the effects of PDE4C on immune therapy, the merged cohort was uploaded into TIDE database (http://tide.dfci.harvard.edu/login/) to calculate the dysregulation score, exclusion score, TIDE score and response rate. *p* < 0.05 was set as a significant cut‐off between high and low PDE4C group.

### 2.15 Statistical analysis

Differences in results were analysed in SPSS 19.0. The differences between the two groups were analysed by unpair test, while multiple group differences were analysed using one‐way analysis of variance analysis combined with LSD‐t analysis. *p* < 0.05 was set as threshold.

## 3 RESULTS

### 3.1 PDE4C and APOC1 were predicted as crucial mediators between M2 macrophage and OS cells

**FIGURE 1:**
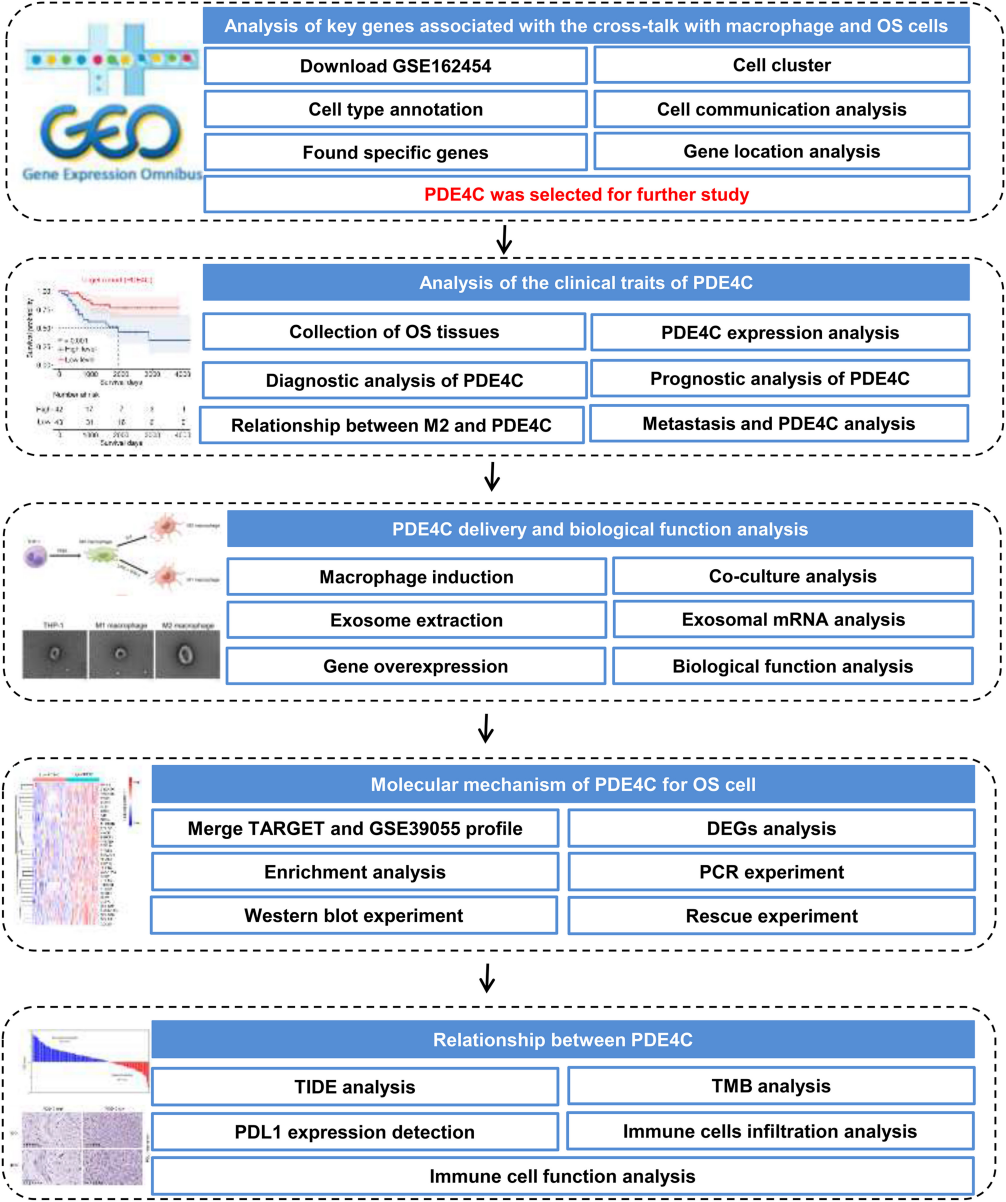
The analysis strategy of this study was exhibited in flowchart.

**FIGURE 2:**
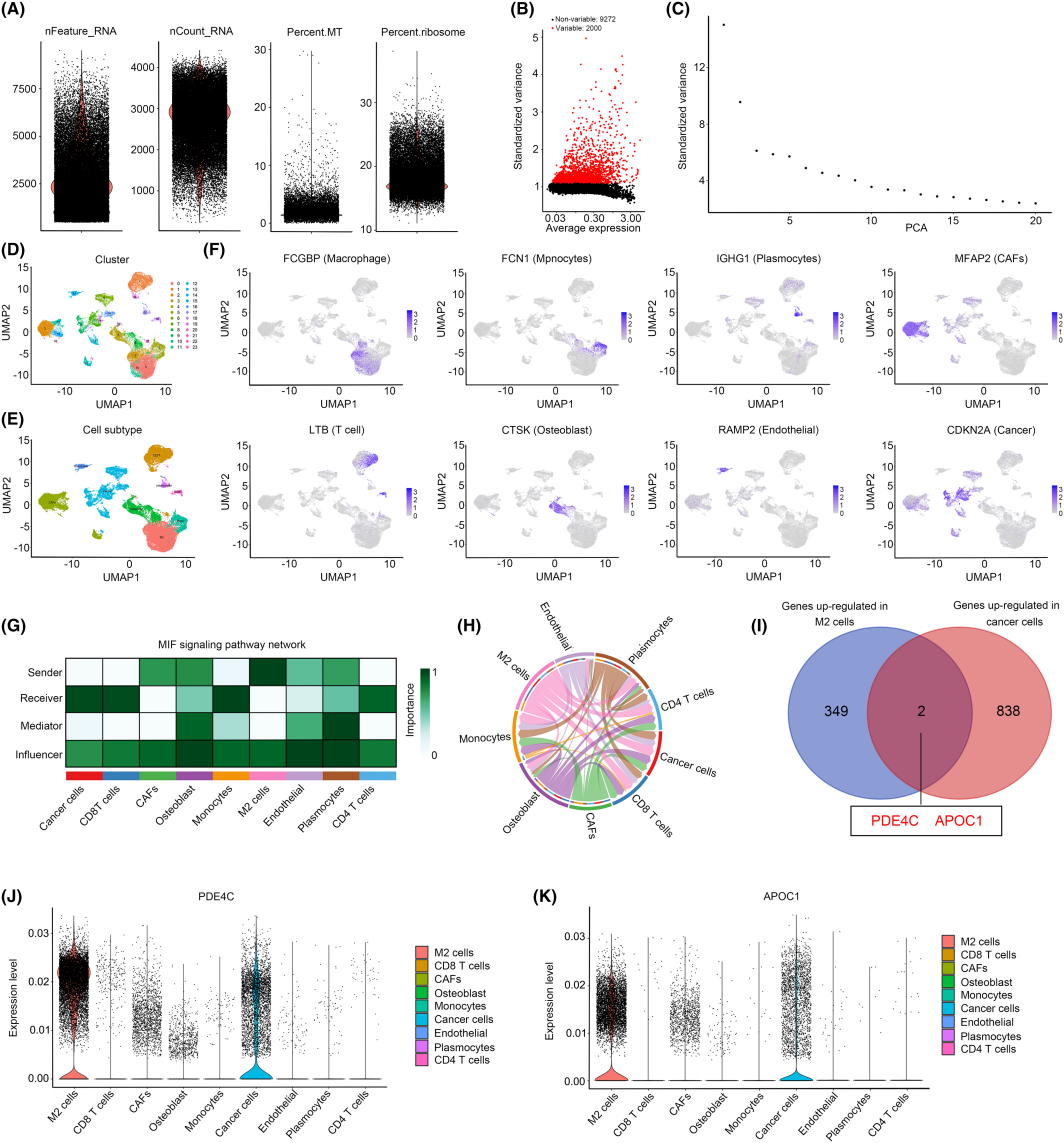
PDE4C and APOC1 were predicted as crucial mediators between M2 macrophage and OS cells. (A) The feature, count, mitochondrial content percentage and ribosome percentage of cells in GSE162454 were computed. (B) Top 2000 genes exhibiting the highest variability were selected. (C) Top 20 PCA was exhibited. (D) The UMAP exhibited 23 cell clusters. (E) The 23 cell clusters were merged into 9 cell types, including CD8 T cells, CD4 T cells, plasmocytes, monocytes, M2 macrophages, osteoblast, endothelial, cancer‐associated fibroblasts (CAFs) and cancer cells. (F) Cell distribution of *FCGBP* (biomarker for macrophage), *FCN1* (biomarker for monocytes), *IGHG1* (biomarker for plasmocytes), *MFAP2* (biomarkers for CAFs), *LTB* (biomarker for T cell), *CTSK* (biomarker for osteoblast), *RAMP2* (endothelial) and *CDKN2A* (biomarkers for cancer). (G) Calculation of the importance of each cell type in cell communication. (H) The cell communication of each cell type. (I) Analysis of genes upregulated in both M2 macrophage and cancer cells. (J) Cell distribution of *PDE4C* was analysed. (K) Cell distribution of *APOC1* was analysed.

Previous research has demonstrated that scRNA‐seq has the potential to identify crosstalk genes between distinct cell types.^12^, ^13^ Consequently, in this study, we have devised an analysis strategy to ascertain the crucial crosstalk genes in M2 macrophages and OS cells (Figure 1). In the initial stage of scRNA sequencing analysis, we computed the feature, count, mitochondrial content percentage and ribosome percentage of the cells included in the study. The cells were selected for further analysis based on the following criteria: feature values ranging from 200 to 7000 and mitochondrial content percentage less than 7 (Figure 2A). Then, the top 2000 genes exhibiting the highest variability in cells were calculated (Figure 2B). After calculating top 20 PCA of these genes (Figure 2C), all of them were used for UMAP cluster analysis, and a total of 23 cell clusters were obtained (Figure 2D). Following cell annotation in CellMarker2.0, these 23 cell clusters were merged into 9 cell types, including CD8 T cells, CD4 T cells, plasmocytes, monocytes, M2 macrophages, osteoblast, endothelial, cancer‐associated fibroblasts (CAFs) and cancer cells (Figure 2E). To ascertain the rationality of cell annotation, an analysis was conducted on the distribution of classical markers of cells, revealing their accuracy (Figure 2F). Furthermore, a cell communication analysis was conducted, revealing a significant level of crosstalk between M2 macrophages and cancer cells. Additionally, M2 macrophages and cancer cells were identified as key senders and receivers, respectively (Figure 2G,H). Following calculating high abundance genes in M2 macrophages and cancer cells, we found that *PDE4C* and *APOC1* was both highly enriched in M2 macrophages and cancer cells (Figure 2I). Through localization analysis, it was determined that M2 macrophages exhibited the highest expression levels of *PDE4C* (Figure 2J) and *APOC1* (Figure 2K), while tumour cells displayed comparatively lower levels. Therefore, we considered that *PDE4C* and *APOC1* were crucial mediators between M2 macrophages and OS cells, while the M2 macrophages serve as the primary origin.

### 3.2 Levels of PDE4C were elevated in OS tissues and associated with poor prognosis and M2 macrophage infiltration

**FIGURE 3:**
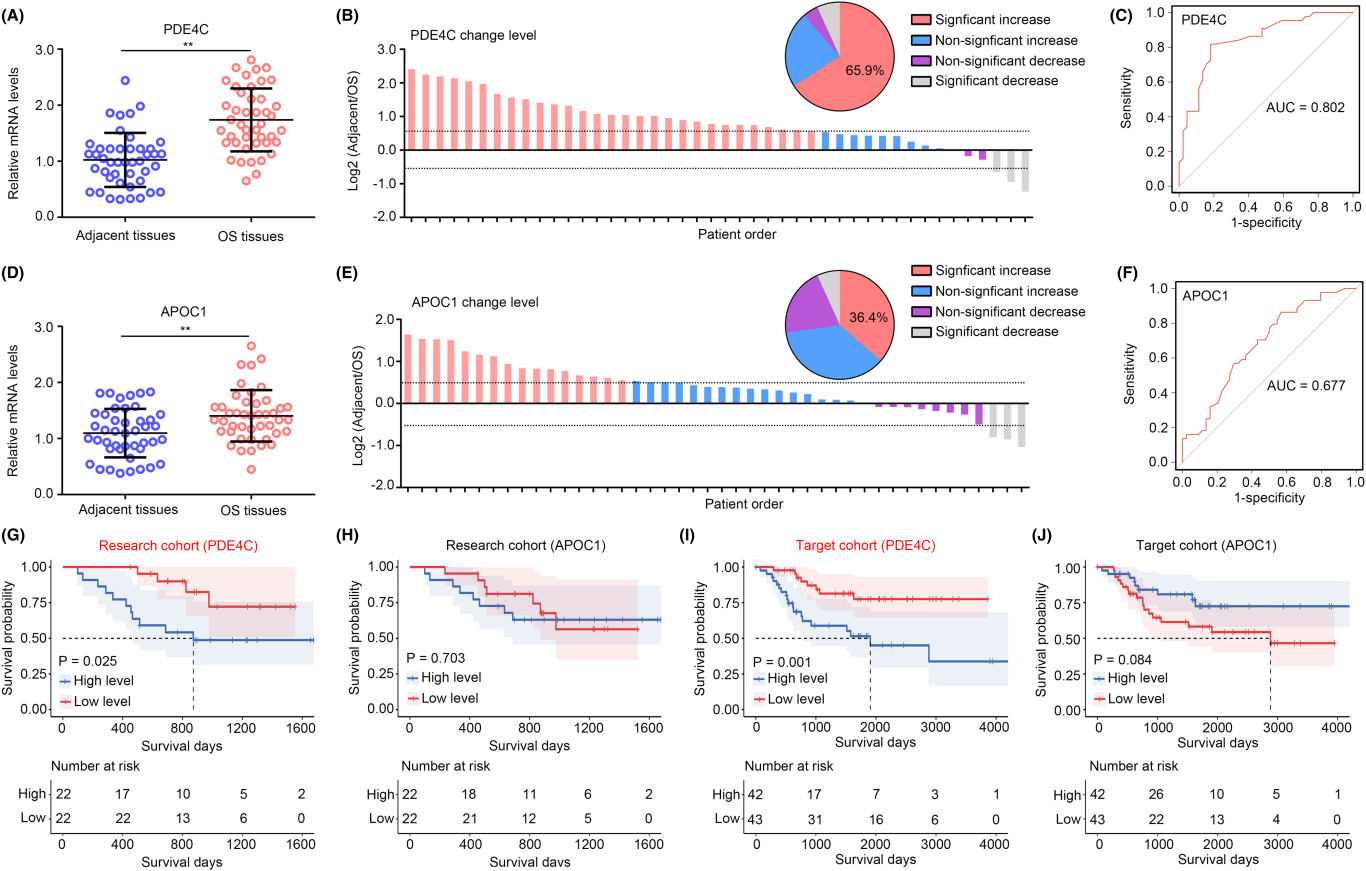
Levels of PDE4C were elevated in OS tissues, and associated with poor prognosis. (A) qRT‐PCR was used to detect the expression of *PDE4C* in adjacent tissues and OS tissues. (B) The change in mRNA levels of *PDE4C* in match adjacent tissues and OS tissues. (C) ROC analysis was performed to determine the diagnostic value of *PDE4C* to distinguish adjacent tissues and OS tissues. (D) qRT‐PCR was used to detect the expression of *APOC1* in adjacent tissues and OS tissues. (E) The change of mRNA levels of *APOC1* in match adjacent tissues and OS tissues. (F) ROC analysis was performed to determine the diagnostic value of *APOC1* to distinguish adjacent tissues and OS tissues. (G and H) Research cohort demonstrated the survival value of *PDE4C* and *APOC1*. (I and J) Target cohort demonstrated the survival value of *PDE4C* and *APOC1*. **p < 0.01 (vs. adjacent tissues).

We then checked the clinical value of PDE4C and APOC1 in OS from our research cohort. High mRNA levels of *PDE4C* were observed in the OS tissues compared with adjacent tissues (Figure 3A). A total of 65.9% OS tissues exhibited a significant increase of *PDE4C* (Figure 3B), and the diagnostic value of *PDE4C* for distinguishing adjacent tissues and OS tissues was 0.802 (Figure 3C). Similarly, we also found that mRNA levels of *APOC1* were also increased in OS tissues (Figure 3D). A total of 36.4% OS tissues exhibited a significant increase of *APOC1* (Figure 3E), and its diagnostic value was 0.677 (Figure 3F). Through performing KM‐plot in our research cohort, high levels of *PDE4C* were found to be associated with poor prognosis (Figure 3G), while *APOC1* had no relationship with prognosis in OS (Figure 3H). These results from our research cohort were consistent with evidences from TARGET cohort (Figure 3I,J). Therefore, we only paid attention on *PDE4C*.

**FIGURE 4:**
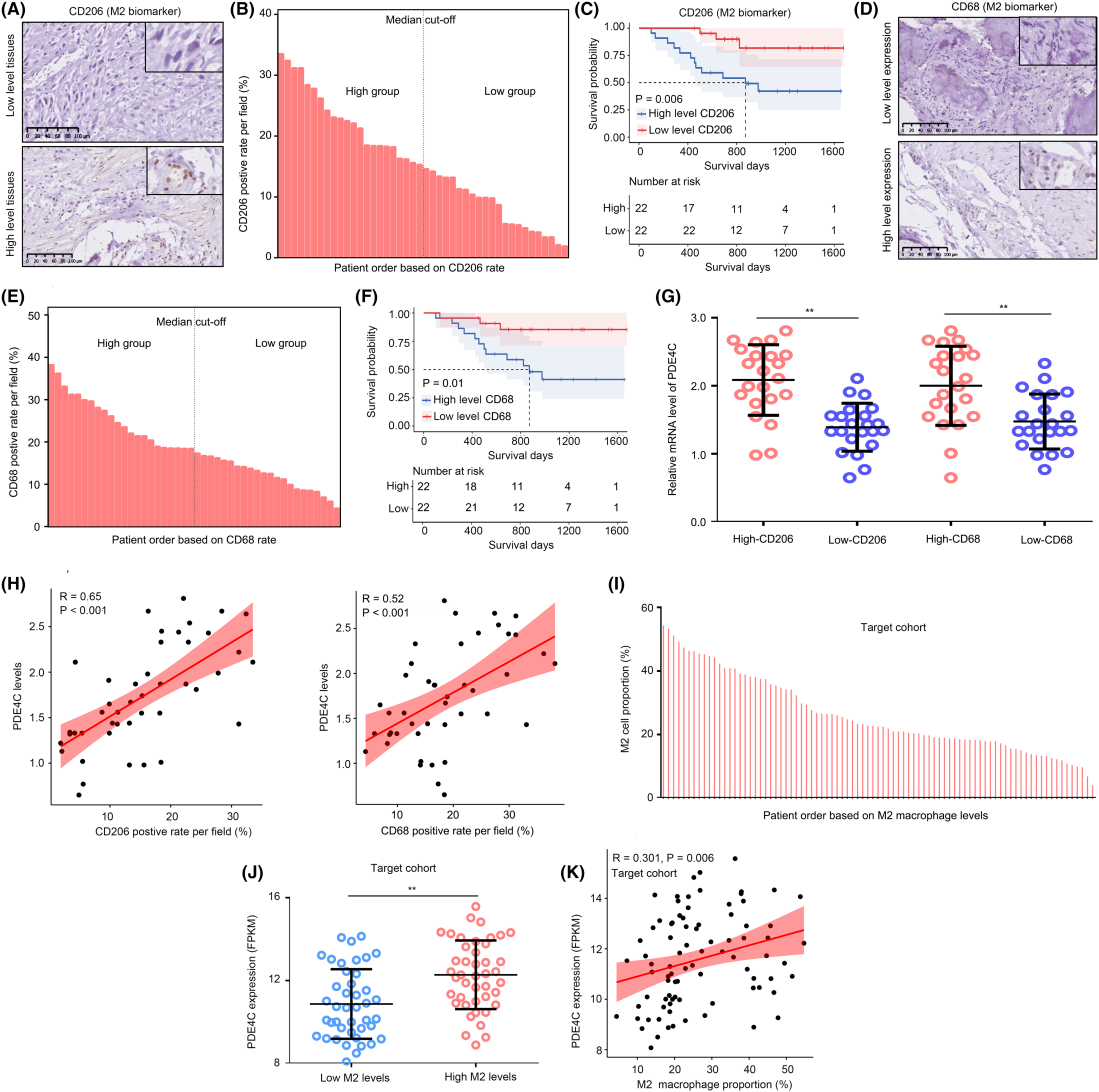
Levels of *PDE4C* were associated with M2 macrophage infiltration. (A) IHC was performed to stain CD206 (M2 biomarker) in OS tissues. (B) OS tissues in research group were clustered into high and low group according to the median value of CD206 expression. (C) The survival difference between high CD206 and low CD206 group. (D) IHC was performed to stain CD68 (M2 biomarker) in OS tissues. (E) OS tissues in research group were clustered into high and low group according to the median value of CD68 expression. (F) The survival difference between high CD68 and low CD68 group. (G) mRNA levels of *PDE4C* in the OS tissues with high and low CD206 or CD68 from our research group. (H) Relationship between *PDE4C* expression and CD206 and CD68 levels in OS tissues from research cohort. (I) Cibersort was used to calculate M2 macrophage levels in OS tissues from TARGET cohort. (J) mRNA levels of *PDE4C* in the OS tissues with high and low macrophage from our TARGET group. (K) Relationship between *PDE4C* expression and M2 macrophage levels from TARGET cohort. **p < 0.01 (high vs. low group).

Moreover, we stained CD206 (Figure 4A) and CD68 (Figure 4D), biomarkers of M2 macrophage in OS tissues, and clustering OS tissues into high and low groups according to the median value (Figure 4B,E). We found that high CD206 and CD68 were both associated with poor prognosis (Figure 4C,F). The results indicated that high M2 infiltration was associated with poor prognosis. Similarly, high mRNA levels of *PDE4C* were observed in the OS tissues with high CD206 and CD68 expression (Figure 4G), and the co‐expression relationship between *PDE4C* and CD206/CD68 levels was 0.65/0.52 (Figure 4H). Moreover, we analysed the relationship between M2 macrophage and PDE4C expression in TARGET cohort. We first used Cibersort to calculate the M2 macrophage level in each OS tissue in TARGET cohort (Figure 4I). Consistent with our research cohort, we found that high expression of *PDE4C* was observed in OS tissues with high M2 macrophage levels (Figure 4J), and the positive co‐relationship between PDE4C and M2 macrophage level was also observed (Figure 4K). This evidence indicated that levels of *PDE4C* were elevated in OS tissues and associated with poor prognosis and M2 macrophage infiltration.

### 3.3 M2 macrophage‐derived PDE4C was associated with metastasis in OS patients

**FIGURE 5:**
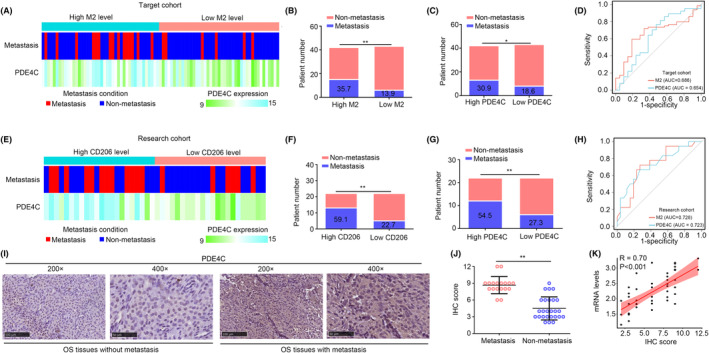
TAM‐derived gene PDE4C was associated metastasis in OS patients. (A) Heatmap exhibited the metastasis condition, M2 macrophage, *PDE4C* expression in OS from TARGET cohort. (B) Metastasis state between OS patients in high M2 macrophage and low M2 macrophage group from TARGET cohort. (C) Metastasis state between OS patients in high *PDE4C* and low PDE4C group from TARGET cohort. (D) Diagnostic value of M2 macrophage and *PDE4C* level for predicting metastasis in OS patients from TARGET cohort. (E) Heatmap exhibited the metastasis condition, M2 macrophage, *PDE4C* expression in OS from research cohort. (F) Metastasis state between OS patients in high M2 macrophage and low M2 macrophage group from research cohort. (G) Metastasis state between OS patients in high *PDE4C* and low PDE4C group from research cohort. (H) Diagnostic value of M2 macrophage and *PDE4C* level for predicting metastasis in OS patients from research cohort. (I and J) IHC was performed to detect the protein levels of PDE4C in OS tissues with and without metastasis. (K) Expression relationship between *PDE4C* mRNA levels and protein levels. *p < 0.05; **p < 0.01 (high vs. low; metastasis vs. non‐metastasis group).

In TARGET cohort, we used Cibersort online tool to analysed the M2 macrophages in OS tissues, and further measured the relationship between metastasis, *PDE4C* expression and M2 macrophage levels. The detailed condition of OS tissues in TARGET cohort was exhibited in a heatmap (Figure 5A). We found that patients in high M2 macrophage group and high *PDE4C* expression group exhibited a metastasis rate in TARGET cohort (Figure 5B,C). The diagnostic values of M2 macrophage and PDE4C expression for predicting metastasis of OS in TARGET cohort were 0.686 and 0.654, separately (Figure 5D). Furthermore, we analysed the relationship between metastasis, *PDE4C* expression and M2 macrophage levels in our research cohort (Figure 5E). Similarly, patients in high CD206 (M2 macrophage) group and high *PDE4C* expression group exhibited more metastasis rate (Figure 5F,G). The diagnostic values of CD206 (M2 macrophage biomarker) and *PDE4C* expression for predicting metastasis of OS in our research cohort were 0.728 and 0.723 (Figure 5H). Through performing IHC, we also found that OS tissues with metastasis had high protein levels of PDE4C (Figure 5I,J). Moreover, we found that the correlation coefficient between PDE4C protein and mRNA levels in OS tissues was 0.70 (Figure 5K). Taken together, M2 macrophage‐derived PDE4C was associated with metastasis in OS patients.

### 3.4 M2 macrophage transmitted *PDE4C* mRNA to OS cells via exosome, and PDE4C had potential to increase cell proliferation and mobility

**FIGURE 6:**
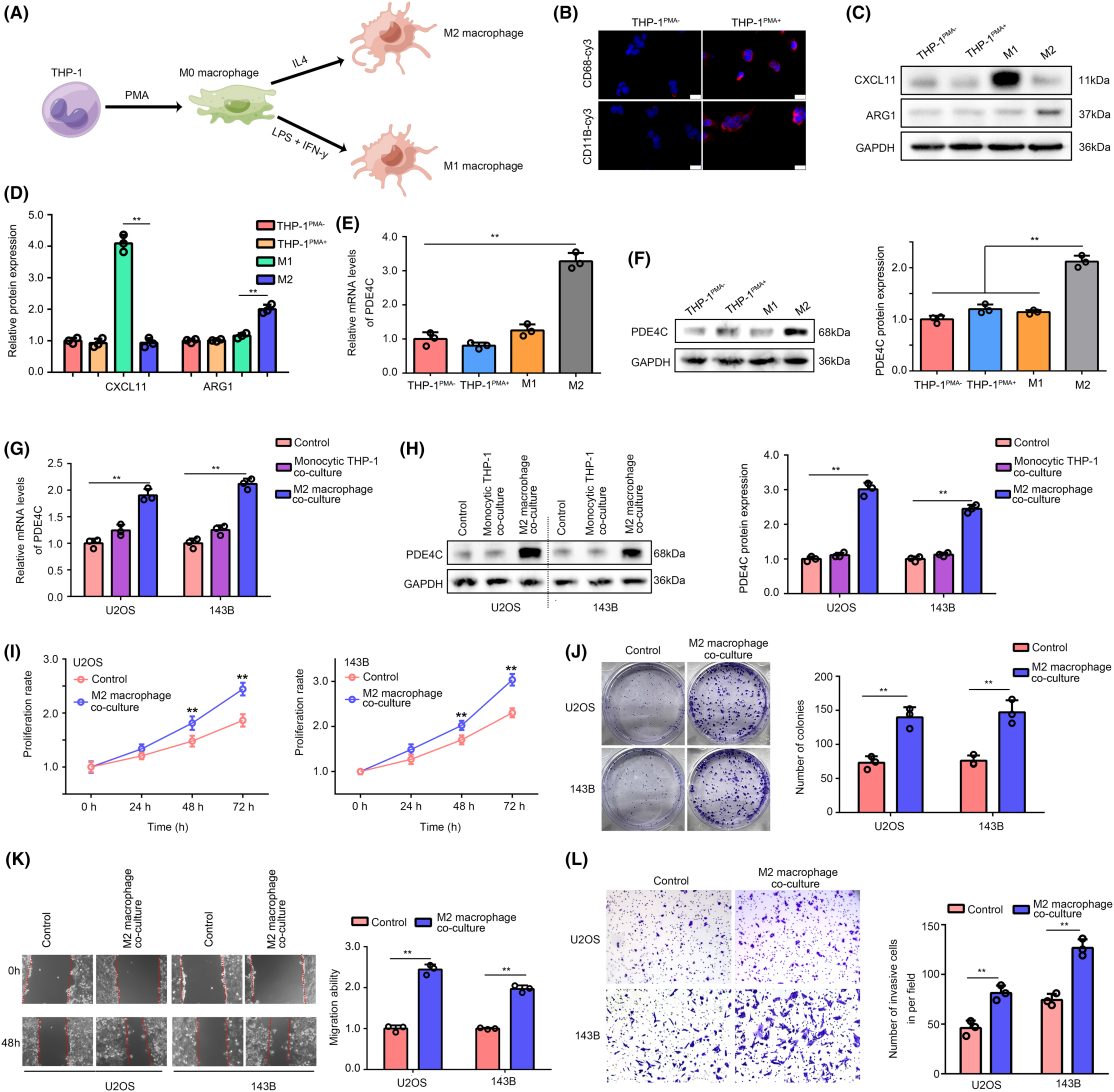
PDE4C expression and cell mobility and proliferation were increased in OS cells after M2 macrophage co‐culture. (A) A diagram exhibited how to induce THP‐1 into M0, M1 and M2 macrophages. (B) Expression of CD68 and CD11B in THP‐1 with and without PMA treatment. White bar mean 20 μm (200×). (C and D) Western blotting was used to detect the expression of CXCL11 and ARG1 in THP‐1, M0 (also named as THP‐1^PMA+^), M1 and M2 macrophages. (E) qRT‐PCR demonstrated the mRNA levels of *PDE4C* in THP‐1, M0, M1 and M2 macrophages. (F) Western blotting demonstrated the protein levels of PDE4C in THP‐1, M0, M1 and M2 macrophages. (G) qRT‐PCR demonstrated the mRNA levels of *PDE4C* in OS cells in control, co‐culturing monocytic THP‐1 and co‐culturing M2 macrophages group. (H) Western blotting demonstrated the protein levels of PDE4C in OS cells in control, co‐culturing monocytic THP‐1 and co‐culturing M2 macrophages group. (I and J) Proliferation and colony formation ability of OS cells in control and co‐culturing M2 macrophages group. (K) Migration ability of OS cells in control and co‐culturing M2 macrophages group. (L) Invasion ability of OS cells in control and co‐culturing M2 macrophage group. **p < 0.01 (line in figures indicated the compare groups).

To explore the effects of M2 macrophage‐derived PDE4C on OS cells, we first developed a strategy to induce monocytic THP‐1 conversing into M0, M1 and M2 macrophage, especially (Figure 6A). To verify the successful transformation from monocytic THP‐1 to M0 macrophage, we used immunofluorescence assay to observe the expression of macrophage biomarker CD68 and CD11B in cells. We found that THP‐1 cells after PMA treatment (THP^PMA+^) had higher CD68 and CD11B expression, indicating successfully inducting monocytic THP‐1 into M0 macrophage (Figure 6B). Then, western blotting was used to analyse the M1‐specific biomarker CXCL11 and M2 specific biomarker ARG1 in cells. In line with the expected idea, M1 macrophage had higher CXCL11 and lower ARG1, whereas M2 macrophage had opposite expression profiles (Figure 6C,D). Interestingly, we found that both the mRNA levels (Figure 6E) and protein levels (Figure 6F) of PDE4C were increased in M2 macrophage compared with monocytic THP‐1, M0 and M1 macrophage.

We then co‐cultured the U2OS and 143B cells with monocytic THP‐1 or M2 macrophage cells in a transwell condition, while cells without co‐culture were set as control. We found that mRNA and protein level of PDE4C were no increased in the U2OS and 143B cells co‐culturing with monocytic THP‐1, whereas mRNA and protein level of PDE4C were elevated in the cells co‐culturing with M2 macrophage (Figure 6G,H). Through performing CCK‐8 and colony formation assay, we found that compared with control group (without M2 co‐culture), OS cells with M2 macrophage co‐culture exhibited higher proliferation and colony formation ability (Figure 6I,J). Furthermore, we used wound healing assays and transwell assays to evaluate the change of migration and invasion of OS cells. It was exhibited that culturing with M2 macrophage significantly increased the migration (Figure 6K) and invasion (Figure 6L) ability of U2OS and 143B cells.

**FIGURE 7:**
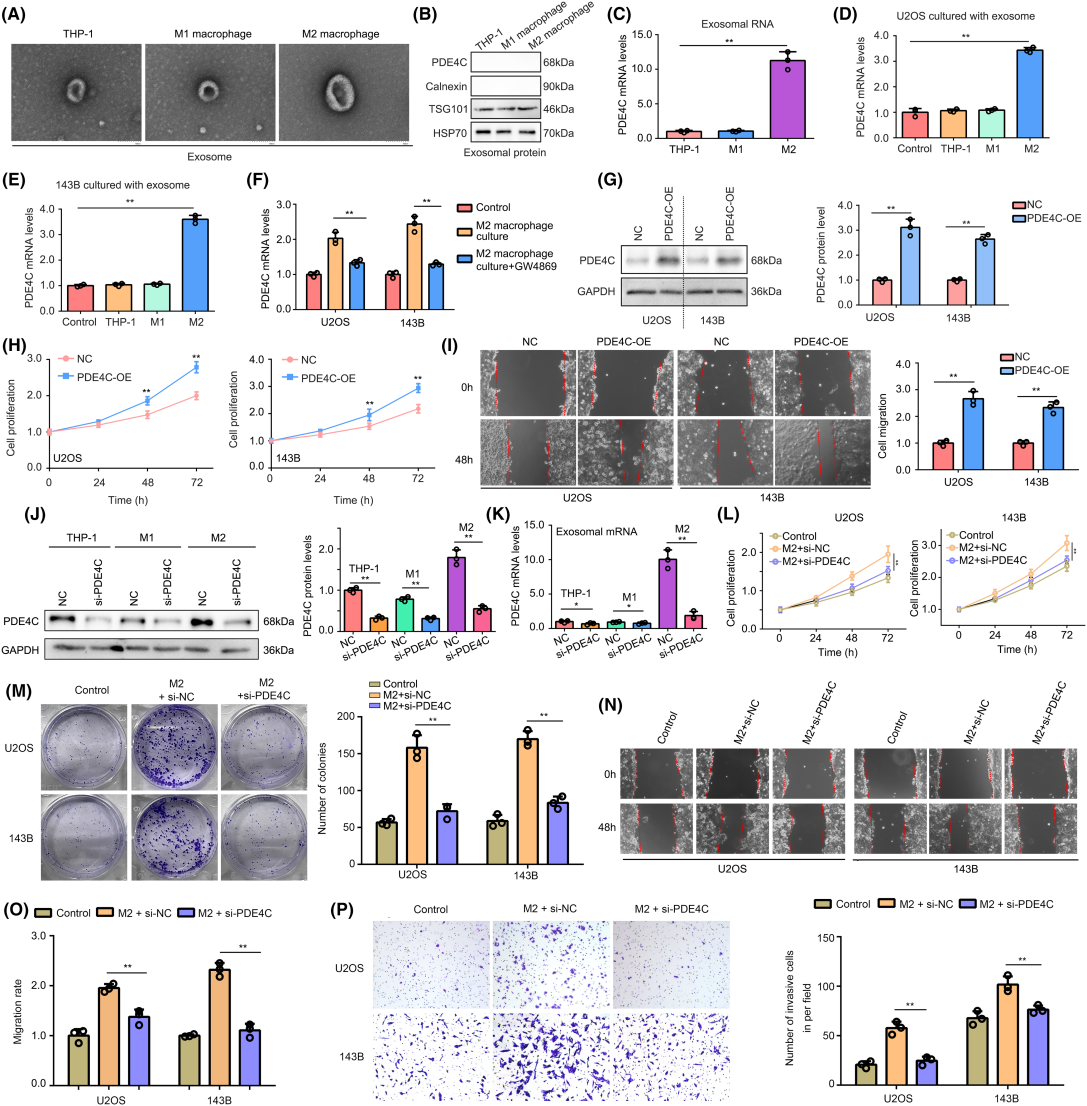
M2 macrophage transmitted *PDE4C* mRNA to OS cells via exosome, and PDE4C had potential to increase cell proliferation and mobility. (A) Typical electron microscope image of exosomes from THP‐1, M1 and M2 macrophage. (B) Expression of PDE4C, Calnexin, TSG101 and HSP70 protein in exosomes from THP‐1, M1 and M2 macrophage. (C) mRNA levels of *PDE4C* in exosomes from THP‐1, M1 and M2 macrophage. (D and E) mRNA levels of *PDE4C* in OS cells after treatment with exosomes from THP‐1, M1 and M2 macrophage. (F) The elevated *PDE4C* levels in OS cells co‐cultured with M2 macrophages could be reversed by pre‐treating the M2 macrophages with GW4869, an inhibitor for exosome formation. (G) Construction of PDE4C‐overexpressed OS cells. (H and I) CCK‐8 and wound healing assay were used to detect the effects of PDE4C overexpression on OS cell proliferation and migration. (J) siRNA was used to knockdown the expression of PDE4C in THP‐1, M1 and M2 macrophages. (K) After knockdown of *PDE4C* in THP‐1, M1 and M2 macrophages, the expression of PDE4C mRNA levels was detected in their exosomes by RT‐qPCR. (L) CCK‐8 was used to detect the proliferation of U2OS and 143B cell in control and culturing M2 macrophage (with or without PDE4C knockdown) group. (M) Colony formation assay was used to detect the colony formation ability of U2OS and 143B cell in control and culturing M2 macrophage (with or without PDE4C knockdown) group. (N and O) Wound healing assay was used to detect the migration ability of U2OS and 143B cell in control and culturing M2 macrophage (with or without PDE4C knockdown) group. (P) Transwell assay was used to detect the invasion ability of U2OS and 143B cell in control and culturing M2 macrophage (with or without PDE4C knockdown) group. *p < 0.05; **p < 0.01 (line in figures indicated the compare groups).

Given the observed increase in mRNA levels of *PDE4C* in OS cells co‐cultured with M2 macrophages, we hypothesized that the transfer of *PDE4C* from M2 macrophages to OS cells is reliant on an mRNA delivery mechanism. To investigate this, we isolated exosomes from THP‐1, M1 and M2 cells, and confirmed their presence using transmission electron microscopy (Figure 7A). Additionally, we employed western blotting with negative biomarker (Calnexin) and positive biomarkers (TSG101 and HSP70) to validate the exosomes (Figure 7B). Interestingly, no PDE4C protein was detected in exosomes from THP‐1, M1 and M2 cells (Figure 7B). However, higher levels of *PDE4C* mRNA were observed in exosomes derived from M2 macrophages compared to those from THP‐1 and M1 cells (Figure 7C). Furthermore, upon culturing exosomes (1 μg) from M2 macrophages, an increase in *PDE4C* mRNA levels was observed in OS cells (Figure 7D,E). Interestingly, the elevated PDE4C levels in OS cells co‐cultured with M2 macrophages could be reversed by pre‐treating the M2 macrophages with GW4869, an inhibitor for exosome formation, for 2 h (Figure 7F). This evidence indicated that M2 macrophage transmitted *PDE4C* mRNA to OS cells via exosome.

To verify the oncogenic effects of *PDE4C* in OS cells, we overexpressed PDE4C in OS cells via transfecting plasmids (Figure 7G). Increased proliferation (Figure 7H) and migration (Figure 7I) were observed in PDE4C overexpression cells. Moreover, we used targeting siRNA to inhibit the expression of *PDE4C* in THP‐1, M1 and M2 macrophages (Figure 7J). We found that PDE4C mRNA in exosomes from M2 macrophages had a significant and dramatic reduction (Figure 7K). Knockdown of *PDE4C* in M2 macrophages significantly reduced the promoted effects of M2 macrophages on proliferation (Figure 7L) and colony formation (Figure 7M) in co‐culture condition. Furthermore, we found that the accelerative effects of M2 macrophage on U2OS and 143B cell migration (Figure 7N,O) and invasion (Figure 7P) can be reversed by *PDE4C* knockdown. PDE4C high‐rich exosomes and PDE4C low‐expressed exosomes were isolated from the medium of NC macrophages and macrophages with sh‐PDE4C. These exosomes (1 μg) were utilized in the treatment of U2OS and 143B cells, revealing a significant increase in PDE4C mRNA levels in cells treated with PDE4C high‐rich exosomes compared to those treated with PDE4C low‐expressed exosomes (Figure S1A). Furthermore, enhanced proliferation and migration were observed in cells treated with PDE4C high‐rich exosomes, but not in those treated with PDE4C low‐expressed exosomes (Figure S1B,C). This evidence demonstrated that M2 macrophage transmitted *PDE4C* mRNA to OS cells via exosome, and PDE4C had potential to increase cell proliferation and mobility.

### 3.5 M2 macrophage‐derived PDE4C increased OS cell proliferation and mobility via upregulating collagens

**FIGURE 8:**
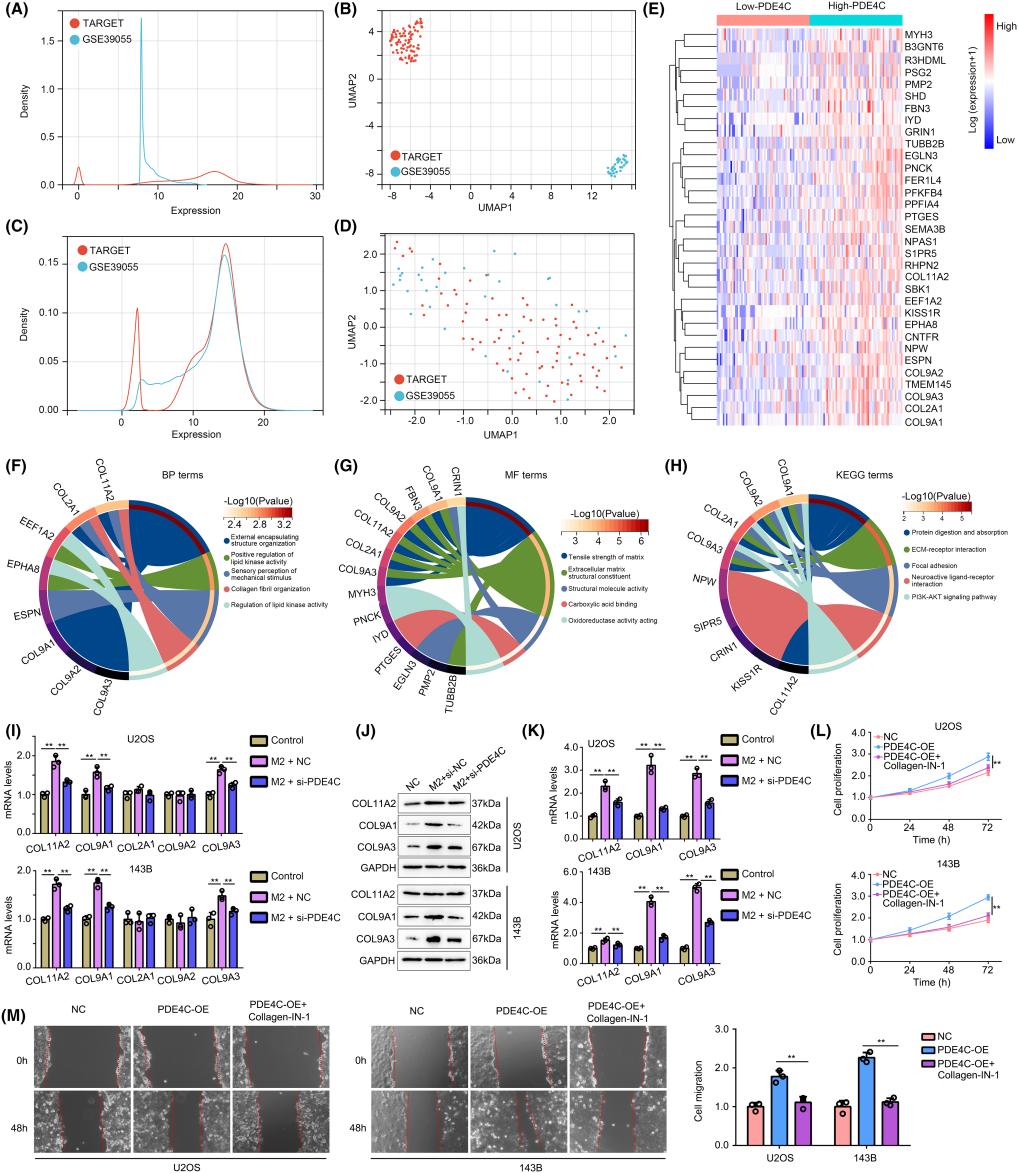
M2 macrophage‐derived *PDE4C* increased OS cell proliferation and mobility via up‐regulating collagens. (A) Expression density of samples in TARGET cohort and GSE39055 cohort before performing batch correction. (B) Expression density of samples in TARGET cohort and GSE39055 cohort after performing batch correction. (C) UMAP density of samples in TARGET cohort and GSE39055 cohort before performing batch correction. (D) UMAP density of samples in TARGET cohort and GSE39055 cohort after performing batch correction. (E) DEGs between the high and low PDE4C in OS tissues from the merged cohort of TARGET cohort and GSE39055 cohort. (F) BP terms of DEGs. (G) MF terms of DEGs. (H) KEGG terms of DEGs. (I) mRNA levels of *COL11A2*, *COL2A1*, *COL9A1*, *COL9A2* and *COL9A3* were detected in OS cells co‐culturing with M2 macrophage after PDE4C knockdown. (J and K) Protein levels of COL11A2, COL2A1, COL9A1, COL9A2 and COL9A3 were detected in OS cells co‐culturing with M2 macrophage after PDE4C knockdown. (L) Effects of collagen inhibitor on OS cell proliferation with PDE4C‐overexpression. (M) Effects of collagen inhibitor on OS cell migration with PDE4C‐overexpression. **p < 0.01 (line in figures indicated the compare groups).

To explore the molecular mechanisms of M2 macrophage‐derived PDE4C in OS cells, we merged and performed batch correction on gene expression profile of TARGET cohort and GSE39055 cohort. The density distribution and UMAP distribution before batch correction were exhibited in Figure 8A,B, while the corresponding density distribution and UMAP distribution after batch correction were exhibited in Figure 8C,D. DEGs between high‐*PDE4C* tissues and low‐*PDE4C* tissues were analysed, and total 33 upregulated DEGs with LogFC≥1 and adjust P value were found (Figure 8E). These 33 upregulated DEGs were enriched in the BP terms such as ‘external encapsulating structure organization’, ‘positive regulation of lipid kinase activity’, ‘sensory perception of mechanical stimulus’, ‘collagen fibril organization’ and ‘regulation of lipid kinase activity’ (Figure 8F). For MF terms, the DEGs were enriched in ‘tensile strength of matrix’, ‘extracellular matrix structural constituent’, ‘structural molecule activity’, ‘carboxylic acid binding’ and ‘oxidoreductase activity acting’ (Figure 8G). KEGG terms of DEGs enriched in were ‘protein digestion and absorption’, ‘ECM‐receptor interaction’, ‘focal adhesion’, ‘neuroactive ligand–receptor interaction’ and ‘PI3K‐AKT signaling pathway’ (Figure 8H). We found that there are five members of collagen including *COL11A2*, *COL9A2*, *COL9A3*, *COL9A1* and *COL2A1* existed in DEGs involving in series of terms associated with mobility process. Therefore, we considered M2 macrophage‐derived PDE4C would affect them. Through performing qRT‐PCR, we found that *COL11A2*, *COL9A1* and *COL9A3* were elevated in both U2OS and 143B cells after co‐culturing with M2 macrophage cells, while knockdown of *PDE4C* in M2 macrophage reversed the effects (Figure 8I). Similarly, the western blotting results indicated that co‐culturing with M2 macrophage cells increased the protein levels of COL11A2, COL9A1 and COL9A3 in OS cells, while knockdown of PDE4C in M2 macrophage reversed the effects (Figure 8J,K). Moreover, we found that treatment with collagen‐IN‐1 (a collagen inhibitor) can significantly reduce the promoted effects of PDE4C on OS cell proliferation (Figure 8L) and migration (Figure 8M). Therefore, we considered that M2 macrophage‐derived PDE4C increased OS cell proliferation and mobility via upregulating collagens.

### 3.6 M2 macrophage‐derived PDE4C can act as a biomarker for predicting the response to immune checkpoint therapy

**FIGURE 9:**
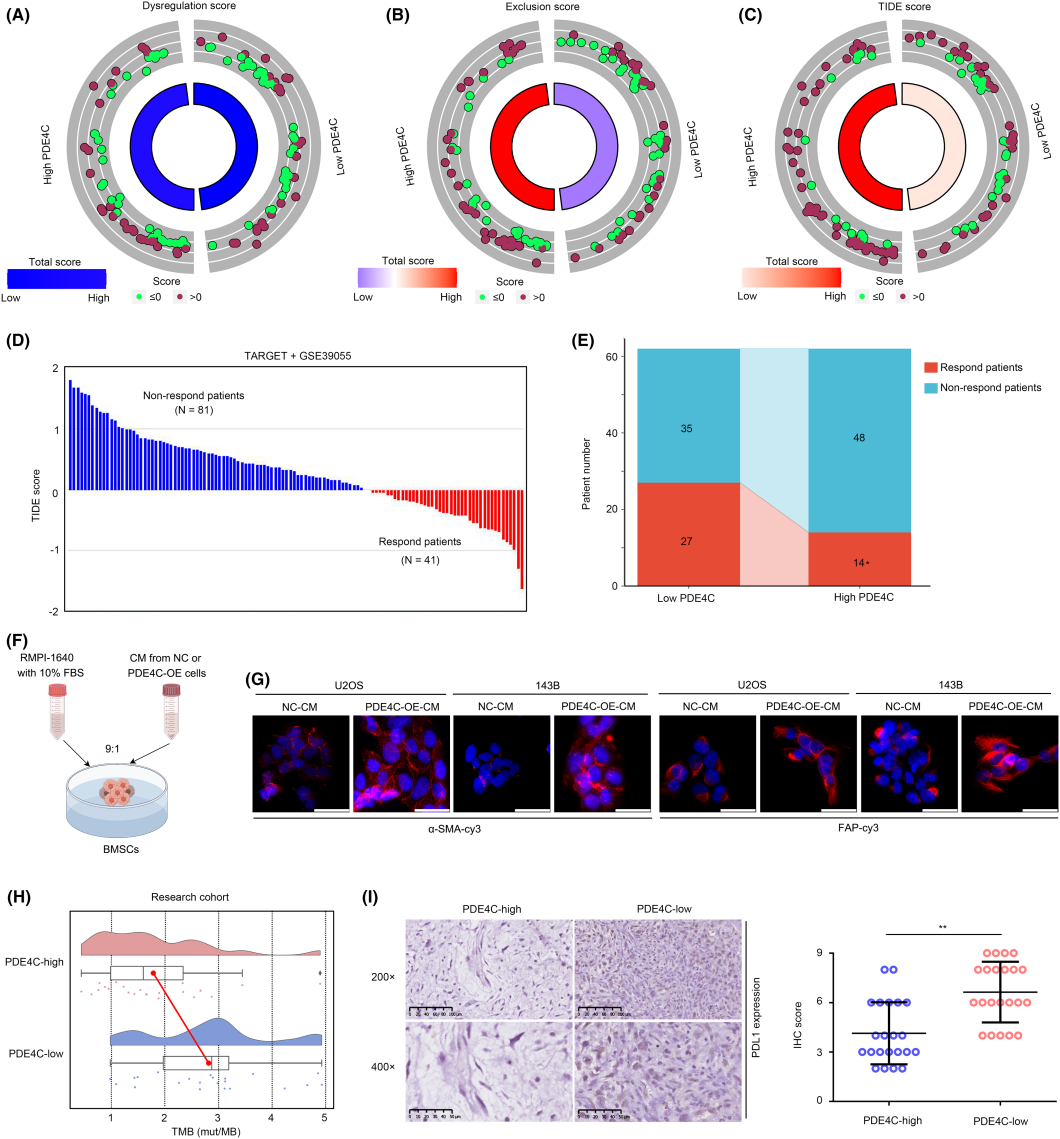
M2 macrophages‐derived PDE4C can act as a biomarker for predicting the respond of immune checkpoint therapy. (A) Dysregulation score in OS tissues with high and low *PDE4C* expression. (B) Exclusion score in OS tissues with high and low *PDE4C* expression. (C) TIDE score in OS tissues with high and low *PDE4C* expression. (D) Respond and non‐respond patients in the merged cohort of TARGET and GSE39055. (E) The differences in response rate between high and low PDE4C group. (F) BMSCs were treated with CM from OS cells with PDE4C‐overexpression or NC cells. (G) α‐SMA and FAP expression in BMSCs after treatment with CM from OS cells with PDE4C‐overexpression or NC cells. White bar mean 50 μm (400×). (H) Difference of TMB in the OS tissues with high and low PDE4C expression group from research cohort. (I) PDL1 expression of TMB in the OS tissues with high and low PDE4C expression group from research cohort. *p < 0.05; **p < 0.01 (high vs. low group).

Macrophage plays a key role in immune regulation, thus affecting the effectiveness of immune therapy. Therefore, we analysed whether M2 macrophage‐derived PDE4C can act as a biomarker for immune therapy. TIDE analysis was conducted, and the dysregulation score was found to have no significant change in low‐*PDE4C* and high‐*PDE4C* group in the total cohort (TARGET + GSE39055) (Figure 9A). However, exclusion score (Figure 9B) and TIDE score (Figure 9C) were significantly increased in OS tissues with high‐PDE4C. We then predicted the respondents and non‐responders in the cohort (Figure 9D). Through statistical analysis, we found that the response rate of immune checkpoint therapy was reduced in the OS patients with high PDE4C (Figure 9E). Previous research has demonstrated that collagens possess the capability to be secreted into the extracellular matrix, thereby functioning as inductors that activate CAFs and facilitate cellular differentiation into CAFs, which had potential to induce immune suppression.^14^, ^15^ Consequently, we procured cell culture medium from OS cells exhibiting overexpression of PDE4C, as well as their corresponding control, and subsequently prepared it as conditioned medium (CM) for the purpose of treating BMSCs (Figure 9F). We found that BMSCs treated with CM from PDE4C‐OE cells exhibited higher α‐SMA and FAP, inducing that PDE4C had the potential to enhance CAF phenotype (Figure 9G). Moreover, we found that high *PDE4C* group OS tissues exhibited low TMB (Figure 9H) and low PDL‐1 expression (Figure 9I). Taken together, results indicated that M2 macrophage‐derived PDE4C can act as a biomarker for predicting the response of immune checkpoint therapy.

## 4 DISCUSSION

TAMs is a group of immune cells infiltrated into tumour tissues, including both M1 and M2 cell populations.^16^ The crosstalk between tumour cells and TAMs is complex. First, TAMs secrete CSF‐1 in a paracrine manner to activate EGF signalling pathway in tumour cells, thereby inducing angiogenesis and distant metastasis of tumour cells.^17^ Second, TAMs secrete various immunosuppressive factors such as TGFβ, IL10 and NO to suppress the anti‐tumour immune response, thus promoting tumour cell proliferation.^18^, ^19^ Finally, TAMs regulate the microenvironment of cancer cells, thus inhibiting the benefit of chemotherapy, target therapy and immune therapy.^20^, ^21^ In OS, previous studies indicated that both M1 and M2 phenotype macrophages were more infiltrated in OS tissues compared with adjacent tissues.^22^ High infiltrated levels of M2 macrophage would reduce the levels of T cells in OS tissues.^23^ Metastatic OS tissues expressed more IL‐34, which can induce monocytes to differentiate into M2 macrophages. Interestingly, after differentiation, M2 macrophages would secrete more IL‐34 to OS cells and activate the ERK pathway in OS cells, thus promoting metastasis.^24^ However, factors involved in the crosstalk between OS cells and TAMs, especially for M2 macrophages were still largely unknown.

ScRNA sequencing is a novel technique that can help to identify cell‐to‐cell interactions and explore specific genes in subtype cells.^25^, ^26^ Herein, we first performed scRNA sequencing analysis and found that M2 macrophage is a key sender in OS tissues, while OS cells played as a key receiver. *PDE4C* and *APOC1* were predicted as key mediators for the crosstalk between M2 macrophage and cancer cells. Interestingly, through exploring their clinical value, we found that levels of *PDE4C* were elevated in OS tissues, and associated with poor prognosis, M2 macrophage infiltration and metastasis case. Therefore, we paid more attention to *PDE4C*.

PDE4C is a member of PDE superfamily and is involved in series of pathways via regulating the metabolism of cAMP.^7^ Dysregulation of PDE family members has been observed in various cancers. Malignant melanoma cells overexpressed PDE1C, while inhibiting PDE1C by vinpocetine obviously reduced malignant melanoma cell proliferation.^27^ High level of PDE3B was observed in colon cancer tissues and associated with cell proliferation.^28^ Therefore, PDEs had been set as therapy targets for cancers. However, the role of PDE4C in OS was still unknown. To further analysing the roles of PDE4C between M2 macrophages and OS cells, experiments were performed. High mRNA and protein levels were found in M2 macrophage compared with monocytic THP‐1, M0 and M1 macrophages. Results may indicate that PDE4C may increase during the differentiation process of M2 macrophage. Furthermore, we found that M2 macrophage cells increased the proliferation and mobility of OS cells, while the knockdown of PDE4C in M2 macrophages can reverse its facilitating effects.

Exosomes, which are extracellular vesicles originating from endocytosis, serve as pivotal regulators of cell signalling. They effectively coordinate autocrine and paracrine functions by transmitting mRNAs, miRNAs, lncRNAs and tsRNAs, thereby modulating the tumour microenvironment and facilitating tumour cell growth and metastasis.^29^, ^30^ Given the observed increase in mRNA levels of *PDE4C* in OS cells co‐cultured with M2 macrophages, we hypothesized that the transfer of *PDE4C* from M2 macrophages to OS cells is reliant on an mRNA delivery mechanism, such as exosome. In accordance with our hypothesis, the presence of *PDE4C* mRNA was observed in exosomes derived from M2 macrophages, while no protein was detected. The application of exosome inhibitors on M2 macrophages prior to co‐culturing with OS cells impeded the upregulation of *PDE4C* mRNA in OS cells. Moreover, promotion effects were observed in OS cells treated with PDE4C‐riched exosomes, but no in those treated with PDE4C low‐expressed exosomes. Collectively, these findings strongly indicate that M2 macrophages transmit *PDE4C* mRNA to OS cells through exosomes.

Collagen (COL) family contains 28 members which are widely distributed in various tissues in human, such as skeletal muscle and skin.^31^ Collagens were expressed in intracellular vesicles, and can be secreted to extracellular matrix in which they regulated the physical characteristics and affected the immune cell infiltration.^32^ Dysregulation of collagens in OS had been reported in previous studies. COL6A1 was highly expressed in OS tissues and promoted OS lung metastasis.^33^ *COL6A3* increased the proliferation and colony formation of OS cells in vitro via activating PI3K/AKT pathway.^34^ *COL1A1* polymorphism at rs1061970 was associated with death cases in patients with OS.^35^ *COL9A3* was involved in the immune regulation and OS progression.^36^ Herein, through analysis of the DEGs between high and low PDE4C OS tissues, we found that five members of collagen including *COL11A2*, *COL2A1*, *COL9A1*, *COL9A2* and *COL9A3* were elevated in OS tissues with high *PDE4C*. qRT‐PCR and western blotting further verified that COL11A2, COL9A1 and COL9A3 were increased in OS cells after co‐culturing with M2 macrophages, while knockdown of PDE4C abolished the effects. Furthermore, inhibition of collagen can reverse the promoted effects of *PDE4C* on OS cell proliferation and mobility. This evidence indicated that M2 macrophage‐derived *PDE4C* may increase OS proliferation and mobility via increasing collagens.

Previous studies indicated that TAMs and collagens were both associated with the response rate of immune therapy.^37^, ^38^ Therefore, we considered that M2 macrophage‐derived *PDE4C* affect the curative effects of immune therapy. TIDE analysis was performed, and we found that high exclusion and TIDE score combined with low response rate were found in the OS tissues with high *PDE4C* expression. Interesting, previous studies indicated that collagens have the ability to be secreted into the extracellular matrix, thus acting as inducers that stimulate the activation of CAFs and promote cellular differentiation into CAFs. Consistent with theory, we found that PDE4C, which mediates the upregulation of collagens, also enhances the CAF phenotype of BMSCs. CAFs play a significant role as immunosuppressive cells within the tumour microenvironment, exerting inhibitory effects on the infiltration of anti‐tumour immune cells, including CD8T, while simultaneously facilitating the immune evasion of tumour cells.^39^, ^40^ This evidence partly explained why OS tissues with high PDE4C expression have higher exclusion scores and TIDE scores. Moreover, OS tissues with high *PDE4C* had lower TMB and lower PDL1 expression. This evidence indicated that TAM‐derived *PDE4C* can act as a biomarker for predicting the response rate after immune therapy.

## 5 CONCLUSIONS

Taken together, M2 macrophage‐derived *PDE4C* can increase the proliferation and mobility of OS cells via increasing collagens, which also can act as biomarkers for predicting patient outcomes and response rate after immune checkpoint therapy. Targeting PDE4C may help the diagnosis and therapy of OS.

## AUTHOR CONTRIBUTIONS

**Feng Pan:** Investigation (lead); methodology (equal). **Runsang Pan:** Investigation (equal); methodology (equal). **Rui Hu:** Investigation (equal); methodology (equal). **Hao Zhang:** Funding acquisition (lead); investigation (equal); methodology (equal). **Shan Lei:** Investigation (equal); methodology (supporting). **Lu Zhang:** Formal analysis (equal); investigation (supporting). **Changhua Zhou:** Data curation (equal); formal analysis (equal); investigation (supporting). **Zhirui Zeng:** Conceptualization (lead); resources (lead); software (lead); writing – original draft (lead). **Xiaobin Tian:** Conceptualization (lead); resources (lead); writing – review and editing (equal). **Quan Xie:** Conceptualization (lead); funding acquisition (equal); resources (lead); supervision (lead).

## FUNDING INFORMATION

The present study was funded by the Department of Science and Technology of Guizhou ([2022]232), the National Natural Science Foundation of China (82060491), The National Natural Science Foundation of China Cultivation Project of the Guizhou Medical University (19NSP957), the Guizhou Medical University High‐level Talent Start‐up Fund Project (J[2021] 001) and Science and Technology Foundation Project of the Guizhou Provincial Health Commission (gzwkj2023‐032).

## CONFLICT OF INTEREST STATEMENT

The authors declare that the research was conducted in the absence of any commercial or financial relationships that could be construed as a potential conflict of interest.

## CONSENT TO PARTICIPATE

Patients enrolled in the study all obtained written informed consent and consent to participate.

## Supporting information


Figure S1.

Table S1.

Appendix S1.

## Data Availability

Routine next‐generation sequencing data and clinical information used for analysis in the present study were downloaded from TARGET database (https://ocg.cancer.gov/programs/target) and GEO database (https://www.ncbi.nlm.nih.gov/geo/) with the index word as GSE39055. Experiment data can be accessed from the corresponding author while it is necessary.
