# Supplementary figures and images for: Analysis of the effects of M2 macrophage‐derived PDE4C on the prognosis, metastasis and immunotherapy benefit of osteosarcoma
Source: J Cell Mol Med. 2024 May 22;28(10):e18395. doi: 10.1111/jcmm.18395 (PMC11109666; doi:10.1111/jcmm.18395)

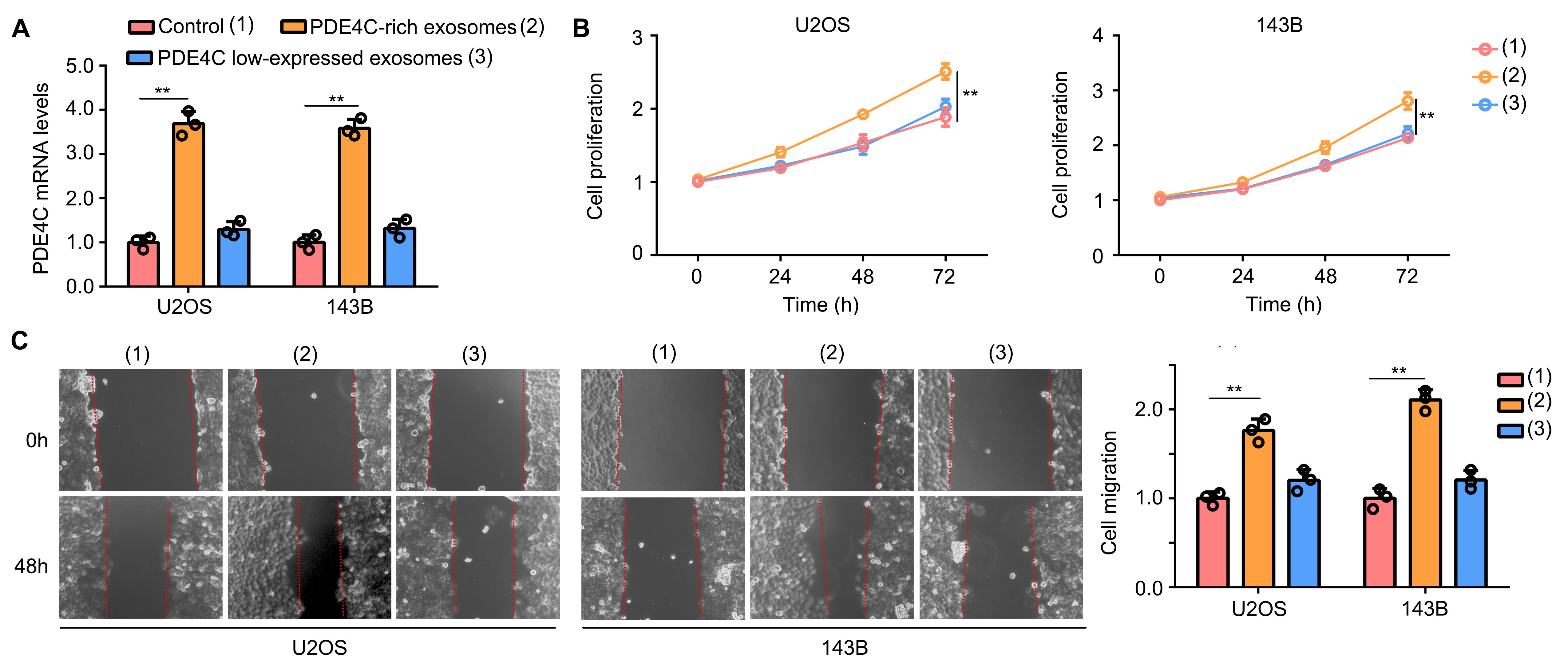

Supplement: Supplementary file 1 — Figure S1. [file JCMM-28-e18395-s002.tif]
