# Supplementary material for: Analysis of the effects of M2 macrophage‐derived PDE4C on the prognosis, metastasis and immunotherapy benefit of osteosarcoma
Source: J Cell Mol Med. 2024 May 22;28(10):e18395. doi: 10.1111/jcmm.18395 (PMC11109666; doi:10.1111/jcmm.18395)
Supplement: Supplementary file 3 — Appendix S1. [file JCMM-28-e18395-s003.docx]

**Supplement figure 1. PDE4C-riched exosomes promoted the proliferation and migration of OS cells.** (A) PDE4C mRNA levels were detected in the OS cells treated with PDE4C rich exosomes and PDE4C low-expressed exosomes. (B) Cell proliferation was detected in the OS cells treated with PDE4C rich exosomes and PDE4C low-expressed exosomes. (C) Cell migration was detected in the OS cells treated with PDE4C rich exosomes and PDE4C low-expressed exosomes. **, P<0.01.
